# Effect of various abiotic stressors on some biochemical indices of *Lepidium sativum* plants

**DOI:** 10.1038/s41598-020-78330-1

**Published:** 2020-12-03

**Authors:** Omar N. Al-Sammarraie, Khalid Y. Alsharafa, Muhamad O. Al-limoun, Khaled M. Khleifat, Sameeh A. Al-Sarayreh, Jehad M. Al-Shuneigat, Hazem M. Kalaji

**Affiliations:** 1 Department of Biological Sciences, Faculty of Science, Mutah University, P.O. Box(7), Mutah, 61710 Jordan; 2 Department of Biochemistry and Molecular Biology, Faculty of Medicine, Mutah University, Mutah, 61710 Jordan; 3 Department of Plant Physiology, Institute of Biology, Warsaw University of Life Sciences SGGW, 159 Nowoursynowska 159, 02-776 Warsaw, Poland

**Keywords:** Plant sciences, Environmental sciences

## Abstract

In this study, the regulation of ascorbate peroxidase (APX) specific activity, anthocyanin, carotenoid, hydrogen peroxide, lipid peroxidation, and protein levels in cress leaves in response to different abiotic stresses were investigated. The total APX specific activity was significantly elevated after 9 days of drought treatment, short-term (2 h) exposure to 10, 100 and 370 µE of light, long-term exposure (at least 6 days) to 100 mM NaCl versus the specific APX activity in the controls. Furthermore, a significant change in total APX activity was detected in response to treatment with different temperatures; this change was an early response to 4 °C and 30 °C for a maximum of 4 h, while short-term exposure to 35 °C did not change total APX activity. The results of the present study revealed that plants have a wide range of mechanisms to cope with different stresses that possibly involve morphological changes. The results indicated that *Lepidium sativum* plants launch common protective pathways only under drought, salinity and high light stresses, while other protective mechanisms/strategies could be responsible for increasing the plants tolerance towards temperature and low light. Future studies will investigate changes in the photosynthetic quantum yield and specific target metabolites, proteins, and nonenzymatic antioxidants.

## Introduction

The nourishing and healing benefits of *Lepidium sativum* (cress), a member of the Brassicaceae family^1^, have been reviewed by Sharma and Agarwal^2^. Cress considered as an important medicinal plant since its seeds and leaves can be used for therapeutic purposes such as inflammation, bronchitis, diuretic, aperient and aphrodisiac properties^2,3^. As long as plants are grown in fluctuating environmental conditions, they must respond to external environmental stimuli. Environmental changes are a source of stress to which plants often respond by the excessive production of oxygen radicals^4–6^.

Different types and severities of abiotic stress and the period of abiotic stress exposure have been studied for their modulation of internal plant homeostasis^7^. Reactive oxygen species (ROS), particularly H_2_O_2_, are signaling molecules that initiate intracellular and systemic signaling or promote oxidative stress and trigger signaling associated with cell death^4,5,7^.

Organisms have developed enzymatic and nonenzymatic systems to act as defensive mechanisms against the effects of ROS to maintain the balance between oxidants and antioxidants for efficient cell functioning. The nonenzymatic system of ROS defense includes tocopherols, glutathione, flavonoids, carotenoids and ascorbic acid^8,9^. While the enzymatic systems includes superoxide dismutases, peroxidases, catalases and enzymes that oxidize or reduce ascorbate such as glutathione reductase, dehydroascorbate reductase and monodehydroascorbate reductase^8,9^. Both systems cooperate to form a complementary mechanism that controls the concentrations of ROS in the organism. Previous attempts on abiotic stress factors^7,10–12^ evaluated different mechanisms by which plants tolerate different levels of various stresses including enzymatic, nonenzymatic and photosynthetic mechanisms such as alteration in pigment content and chlorophyll fluorescence. Ascorbate peroxidases (APXs) are a group of antioxidant enzymes in plants. All the forms of APX are thought to function as scavengers of H_2_O_2_, which is generated continuously in cells^13^. Superoxide anion (O_2_^−^) is formed in the chloroplasts of photosynthetic organisms and in mitochondria by reactions of the electron transport chain. In both cases, superoxide dismutase converts O_2_^−^ to H_2_O_2_, which can then be removed by APX or catalase^6^. Various environmental stimuli, such as drought, salt stress, high light levels, high and low temperatures, H_2_O_2_ and abscisic acid, modulate the expression of APX*-*encoding genes^14^.

This study aimed to identify changes in certain biochemical parameters such as APXs (APX; EC 1.11.1.7), which play a protective role, as well as the levels of nonenzymatic antioxidants, including anthocyanins and carotenoids, in *L. sativum* leaves under different abiotic stress conditions. Moreover, this study intended to investigate the effects of various abiotic stresses on the levels of hydrogen peroxide, proteins and lipid peroxidation in *L. sativum* leaves.

## Materials and methods

### Plant materials and growth conditions

*L. sativum* seeds (Vilmorin, France) were obtained from local distributer and germinated in Molecular Biology Research Laboratory/ Department of Biological Sciences/ Mutah University, Jordan. The germinated seeds grown under controlled conditions (14 h under ~ 54 µE light at 21 °C/10 h in dark at 20 °C; 55–60% relative humidity) then transplanted to 2/1/1 (vol/vol/vol) mixture of peat moss, perlite and vermiculite. After 6 weeks of growth, plants were either subjected to one of the abiotic stress treatments for different time periods or kept in the plant growth chamber under the previously specified controlled conditions as control experiments.

### Abiotic stress treatments

To induce abiotic stress, 6-week-old growing seedlings were used in abiotic stress treatments as designed previously^7^. For drought treatment, plants were introduced to a water deficit by withholding water for 3, 6 or 9 days. Control samples were irrigated continually three times a week for the same time periods (3, 6 or 9 days). For salinity treatment, 6-week-old plants were irrigated with a 100 mM NaCl solution three times per week for up to 14 days. Interleaves samples were collected at 2, 6, 10 and 14 days of treatment. Control samples were irrigated with tap water three times a week for an additional 2, 6, 10 and 14 days under controlled growth conditions. Heat shock was imposed by transferring the 6-weeks plants to a growth chambers adjusted at 4, 25, 30 or 35 °C. Samples were collected from interleaves after 2, 4 and 6 h of incubation at the studied temperatures. In order to evaluate the impact of light quantity, 6-week-old plants were grown under light at ≈54 μmol photon m^2^ s^−1^ (control); then, plants subjected to light quantity treatment under 10 μmol photon m^−2^ s^−1^, 100 μmol photon m^−2^ s^−1^, or 370 μmol photon m^−2^ s^−1^ of light. Samples were collected from interleaves after 2, 6, 12 and 24 h of growth under the studied light quantity. At the end of each specific abiotic treatment, leaves collected from the treated plants were directly frozen in liquid nitrogen and stored at − 80 °C until further analysis. Untreated control plants for all four-stress conditions were grown in parallel with the treated plants.

### Quantification of anthocyanin and carotenoid contents

Twenty mg of the frozen leaves, treatment and control experiments, were grinded into fine powder in liquid nitrogen. Anthocyanin and carotenoid pigments were extracted from the grinded tissue using 1 mL of cold buffer containing methanol/HCl/water (90/1/1, vol/vol/vol). After homogenization, the suspension was incubated for 1 h in the dark. The samples were centrifuged for 15 min at 16,240 × *g*. The methods of Sims and Gamon^15^ were used to spectrophotometrically quantify the pigments.

### H_2_O_2_ assay

A total of 100 mg of frozen leaf material was lysed with 0.1% (wt/vol) trichloroacetic acid, followed by centrifugation at 15,000 × *g* for 15 min at 4 °C. Then, 0.5 mL supernatant was added to 1.5 mL of assay solution composed of 0.5 mL of 10 mM potassium phosphate buffer (pH 7.0) and 1 mL of 1 M KI, mixed gently and the absorbance of the assay mixture was read at 390 nm^16^.

### Lipid peroxidation assay

Aerial plant tissue (50 mg) was lysed in 1 mL 80% (vol/vol) ethanol on ice. The homogenate was centrifuged at 16,000 × *g* for 20 min at 4 °C. The supernatant (0.5 mL) was mixed with 0.5 mL 20% (w/v) trichloroacetic acid (TCA) containing 0.65% (wt/vol) thiobarbituric acid (TBA). After incubation at 95 °C for 30 min, the reaction was immediately cooled in an ice bath. Following centrifugation at 10,000 × *g* for 10 min, the absorbance of the supernatant at 532 and 600 nm was measured. The absorbance value at 600 nm, indicating nonspecific absorption, was subtracted from the absorbance of the supernatant at 532 nm to determine the level of malondialdehyde (MDA) as final product of lipid peroxidation^17^. The MDA concentration was calculated with an extinction coefficient of 155 mM^−1^ cm^−1^.

### Proteins quantification

Protein was extracted from aerial tissue (50 mg) using 1 mL 100 mM HEPES buffer (pH 7.6) on ice and centrifuged at 16,240 × *g* for 10 min at 4 °C. The protein content of 0.2 mL supernatant was determined following Bradford method^18^. Bradford reagent (1 ml) was added to the supernatant, and the absorbance of the mixture at 595 nm was measured.

### APX specific activity

Aerial plant tissue (50 mg) was extracted in 125 µL 100 mM HEPES–NaOH (pH 7.6). The homogenate was centrifuged at 16,240 × *g* and 4 °C for 5 min. Crude extract (50 µL) was added to 1 mL of the assay solution composed of 50 mM HEPES–NaOH buffer (pH 7.6), 50 µL of 5 mM ascorbate and 100 µl of 3 mM H_2_O_2_ to initiate the reaction. The absorbance of the reaction mixture at 290 nm was measured spectrophotometrically. The APX activity was standardized according to the protein content^9^.

### Statistical analysis

Samples were analyzed in triplicate in all experiments, and all the assays were carried out in triplicate. The results expressed as the mean ± SD. The results of each analysis were compared using analysis of variance (ANOVA) with Tukey’s HSD, differences between exposure treatments and the corresponding controls were considered statistically significant if *P* < 0.05.

## Results

### Anthocyanin and carotenoid contents

**Figure 1:**
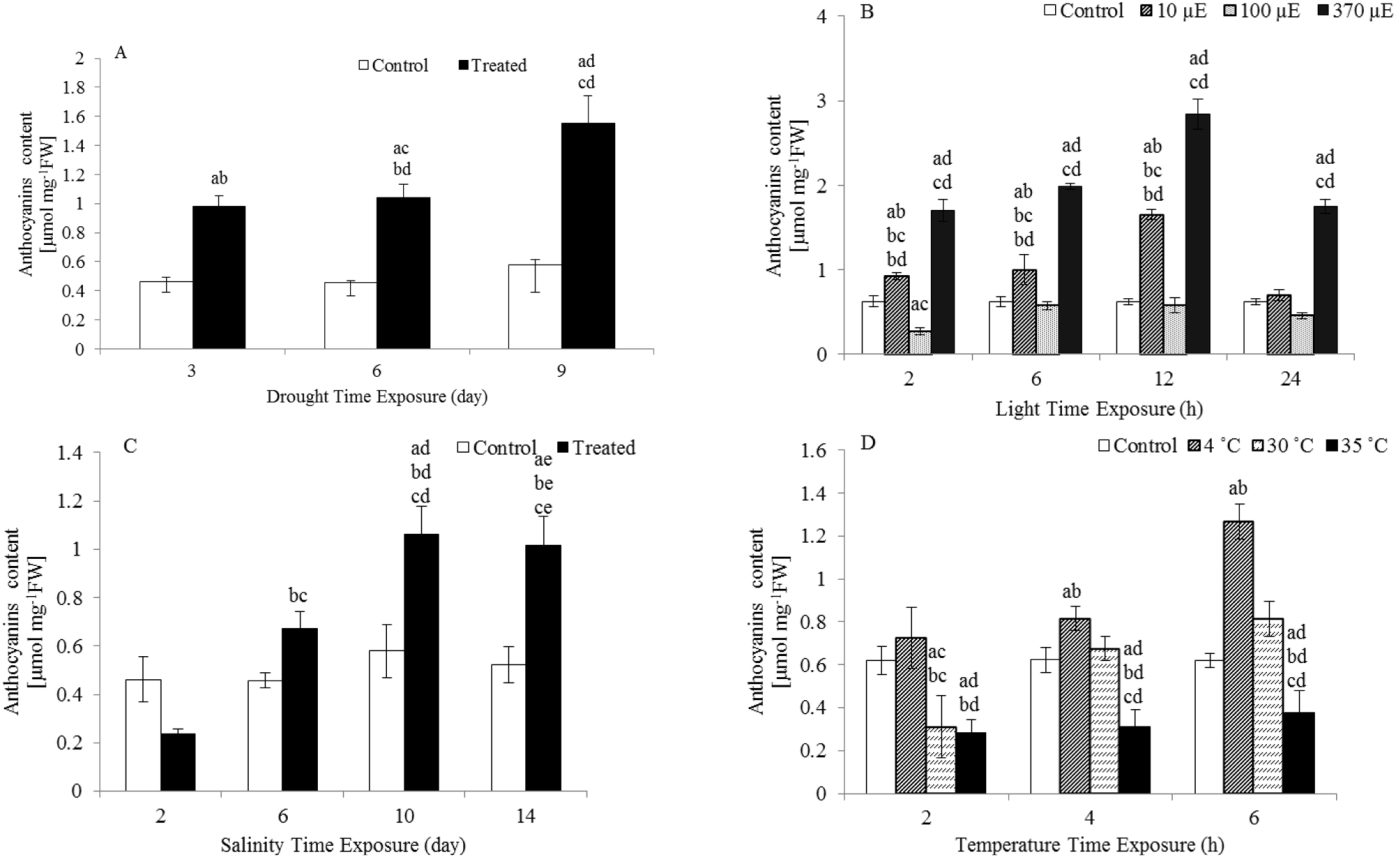
Anthocyanin content in the leaves of *L. sativum* subjected to various abiotic stresses. (**A**) Drought. (**B**) Light intensity. (**C**) Salinity. (**D**) Heat. The effect of different abiotic stresses on anthocyanin content at different time points compared with the anthocyanin content of controls. Data represent mean values ± SD; n = 3. The significance of the differences was calculated using Tukey’s test, with *P* < 0.05 indicating a significant difference.

In this study, the anthocyanin content changed immediately after exposure to different abiotic factors. There were significant increases in the anthocyanin content following drought and light quantity treatments. Six and nine days of drought treatment induced a 2.2- and 2.6-fold increase in anthocyanin content, respectively. Exposure to 10 µE light for 12 h caused a 2.7-fold increase in anthocyanin content, and exposure to 370 µE light for 2, 6, 12 and 24 h increased the anthocyanin content 2.7-, 3.2-, 4.6- and 2.8-fold, respectively, compared to the anthocyanin content of the controls (Fig. 1).

**Figure 2:**
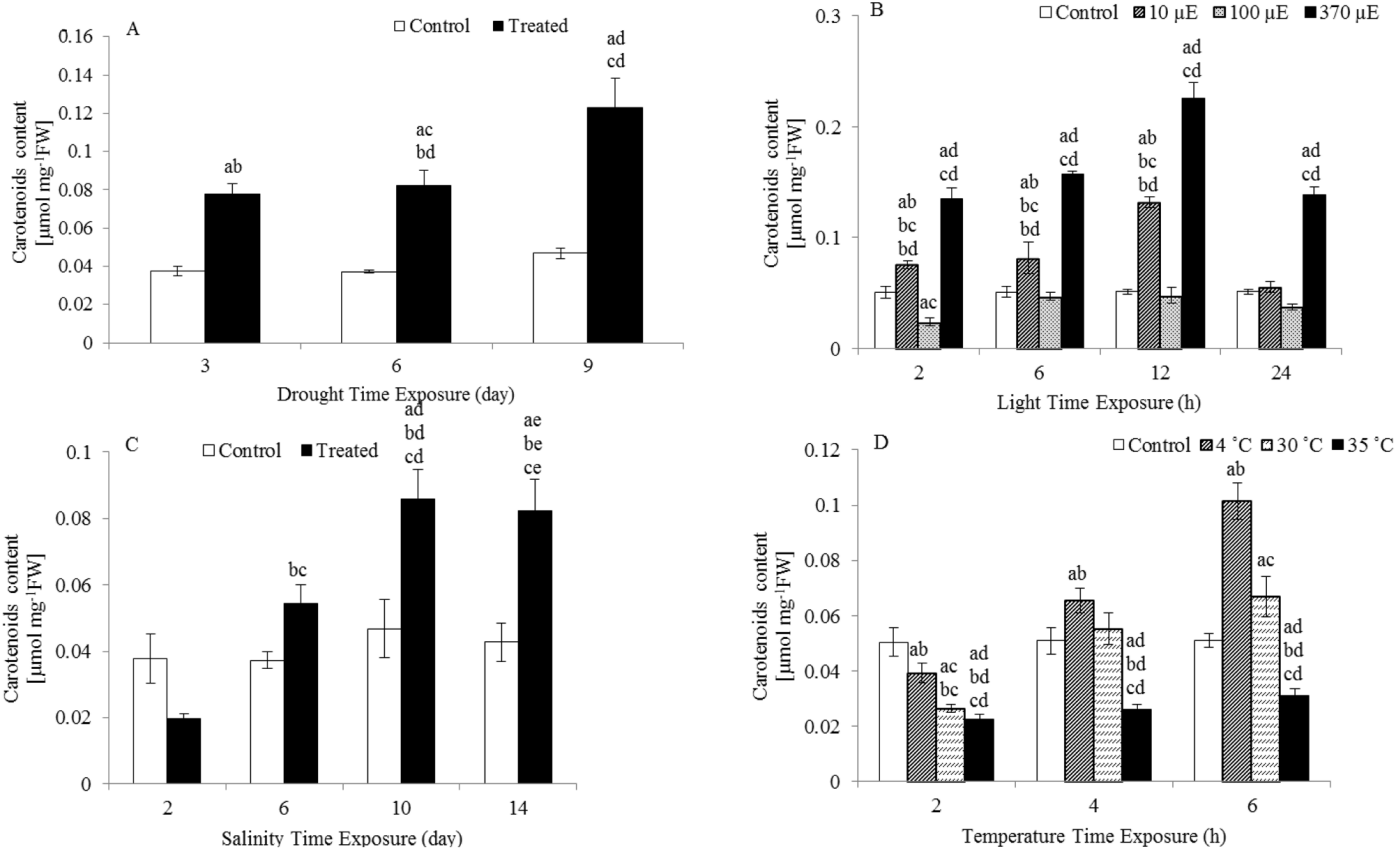
Carotenoid content in the leaves of *L. sativum* subjected to various abiotic stresses. (**A**) Drought. (**B**) Light intensity. (**C**) Salinity. (**D**) Heat. The effect of different abiotic stresses on carotenoid content at different time points compared with the carotenoid content of controls. Data represent mean values ± SD; n = 3. The significance of the differences was calculated using Tukey’s test, with *P* < 0.05 indicating a significant difference.

In parallel to the increased anthocyanin content, the carotenoid content was significantly higher in response to the same abiotic factors versus the carotenoid content of the controls (Fig. 2).

### Hydrogen peroxide accumulation

**Figure 3:**
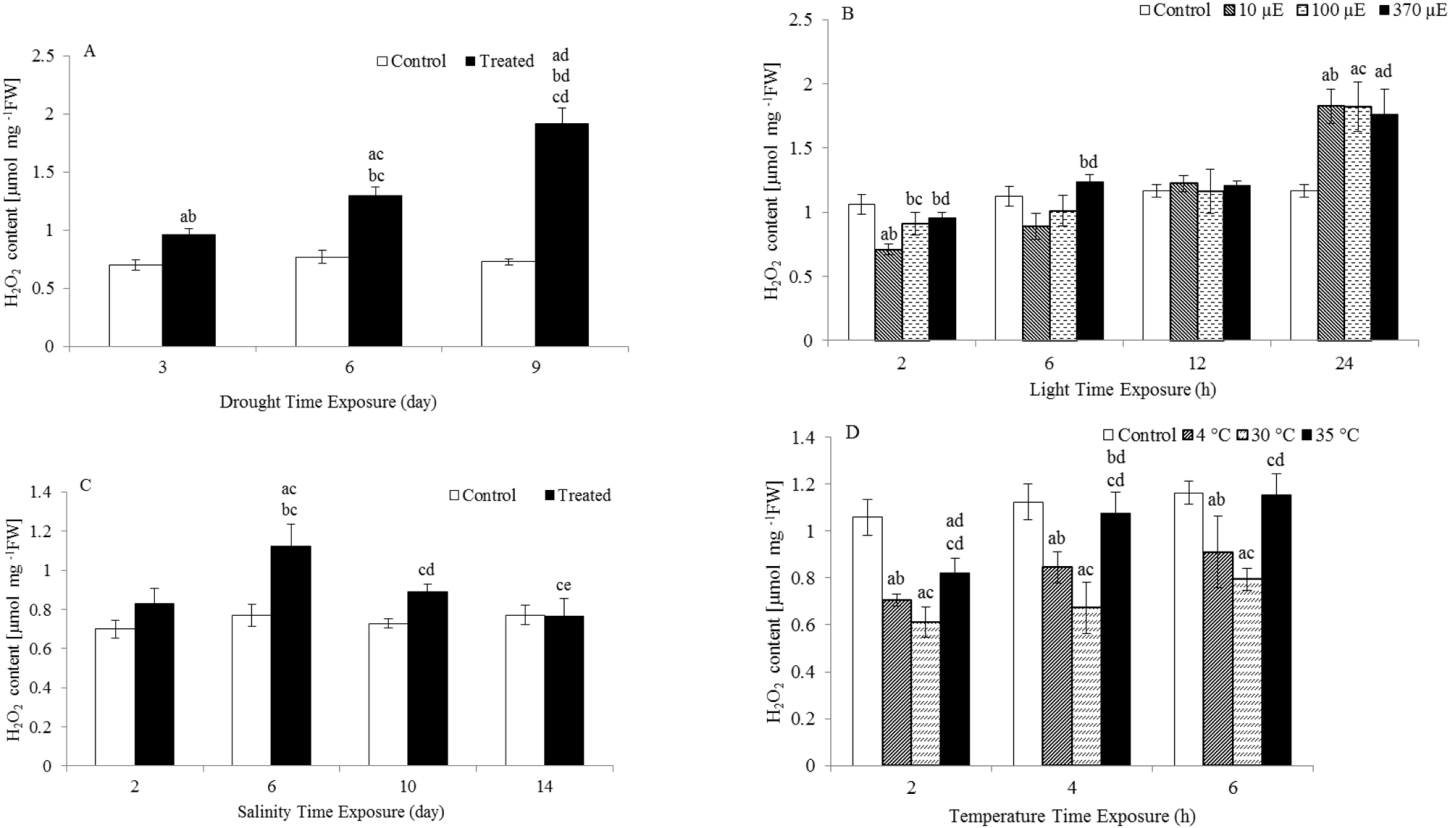
H_2_O_2_ content in the leaves of *L. sativum* subjected to various abiotic stresses. (**A**) Drought. (**B**) Light intensity. (**C**) Salinity. (**D**) Heat. The effect of different abiotic stresses on H_2_O_2_ content at different time points compared with the H_2_O_2_ content of controls. Data represent mean values ± SD; n = 6. The significance of differences was calculated using Tukey’s test, with *P* < 0.05 indicating a significant difference.

Figure 3 shows the effects of different abiotic factors on H_2_O_2_ content in the examined cress leaves. H_2_O_2_ levels in the leaves increased depending on the type and duration of abiotic stress treatment. The H_2_O_2_ levels were significantly higher following drought, light quantity and salinity treatment. The H_2_O_2_ levels in drought-treated plants were 1.4-, 1.7- and 2.6-fold higher than those in well-watered plants after 3, 6 and 9 days of treatment, respectively. However, significant changes in H_2_O_2_ production after 24 h exposure to all light quantities were observed. After 24 h of exposure to 10 µE, 100 µE and 370 µE light, the H_2_O_2_ level peaked and was 1.6-fold higher than the H_2_O_2_ level in control plants. The H_2_O_2_ levels increased in salinity-treated plants by 1.5-fold and 1.2-fold compared to the H_2_O_2_ levels in control plants after 6 and 10 days of treatment, respectively.

### Effects on lipid peroxidation level

**Figure 4:**
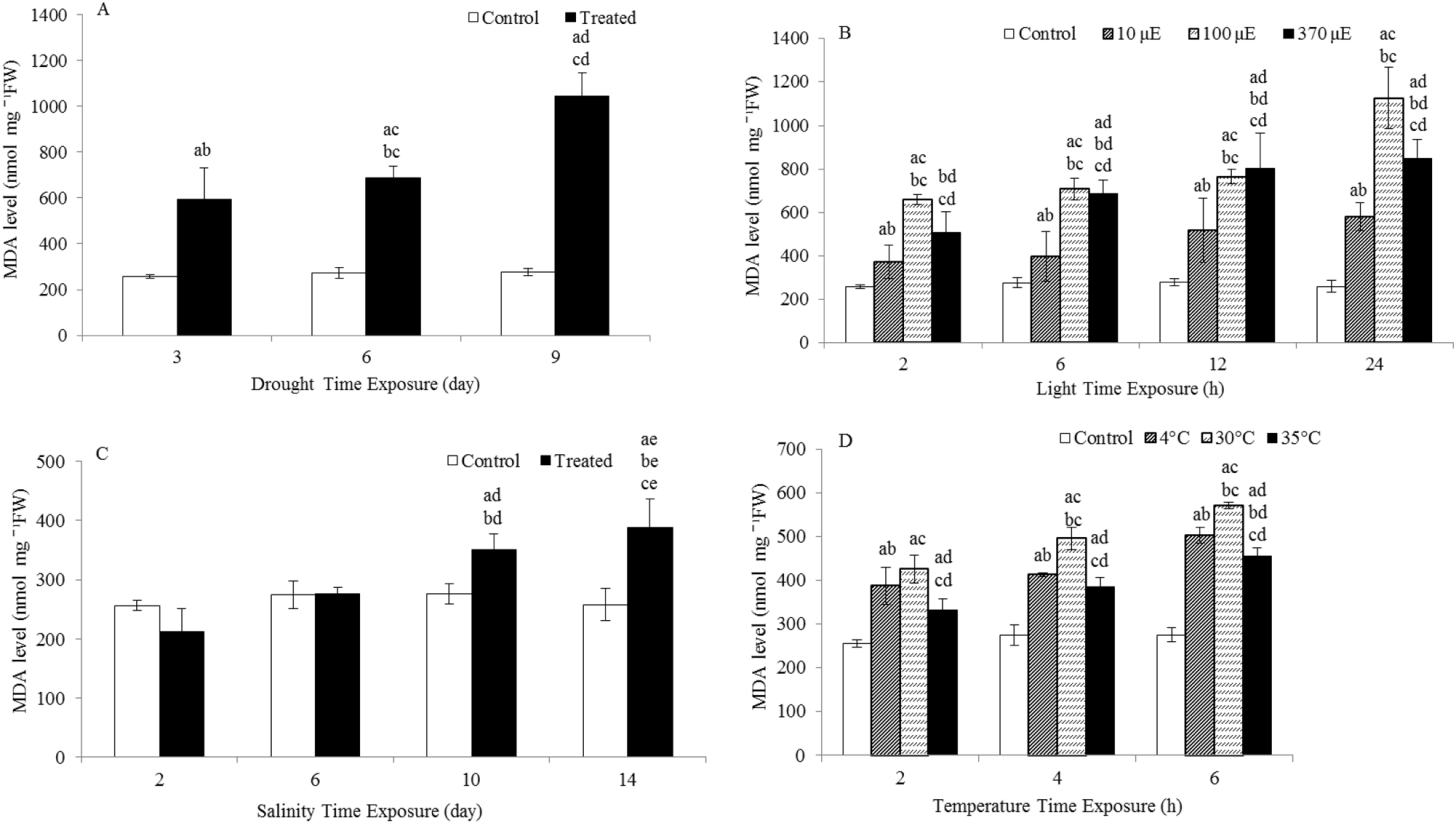
Lipid peroxidation levels (MDA levels) in the leaves of *L. sativum* subjected to various abiotic stresses. (**A**) Drought. (**B**) Light intensity. (**C**) Salinity. (**D**) Heat. The effect of different abiotic stresses on lipid peroxidation levels at different time points compared with lipid peroxidation levels in the controls. Data represent mean values ± SD; n = 3. The significance of differences was calculated using Tukey’s test, with *P* < 0.05 indicating a significant difference.

Given that drought, light and salinity stresses induced H_2_O_2_ production, experiments were carried out to examine whether this increase in H_2_O_2_ was related to oxidative damage in the leaves. For this purpose, the MDA level was analyzed (Fig. 4). MDA levels were significantly increased by 2.3-, 2.5- and 3.8-fold after 3, 6 and 9 days of drought treatment, respectively. However, treatment with 10 µE, 100 µE and 370 µE light for 24 h significantly increased the levels of MDA, which peaked and was 2.2-, 4.4- and 3.3-fold greater than the MDA level in control plants, respectively. Additionally, the MDA levels in salinity-treated plants were significantly increased by 1.3- and 1.5-fold after 10 and 14 days of treatment, respectively, compared to the MDA levels in the controls. The MDA levels remained very low and significantly decreased in the temperature-treated plants compared to those in the control plants.

### Effects on the total protein content

**Figure 5:**
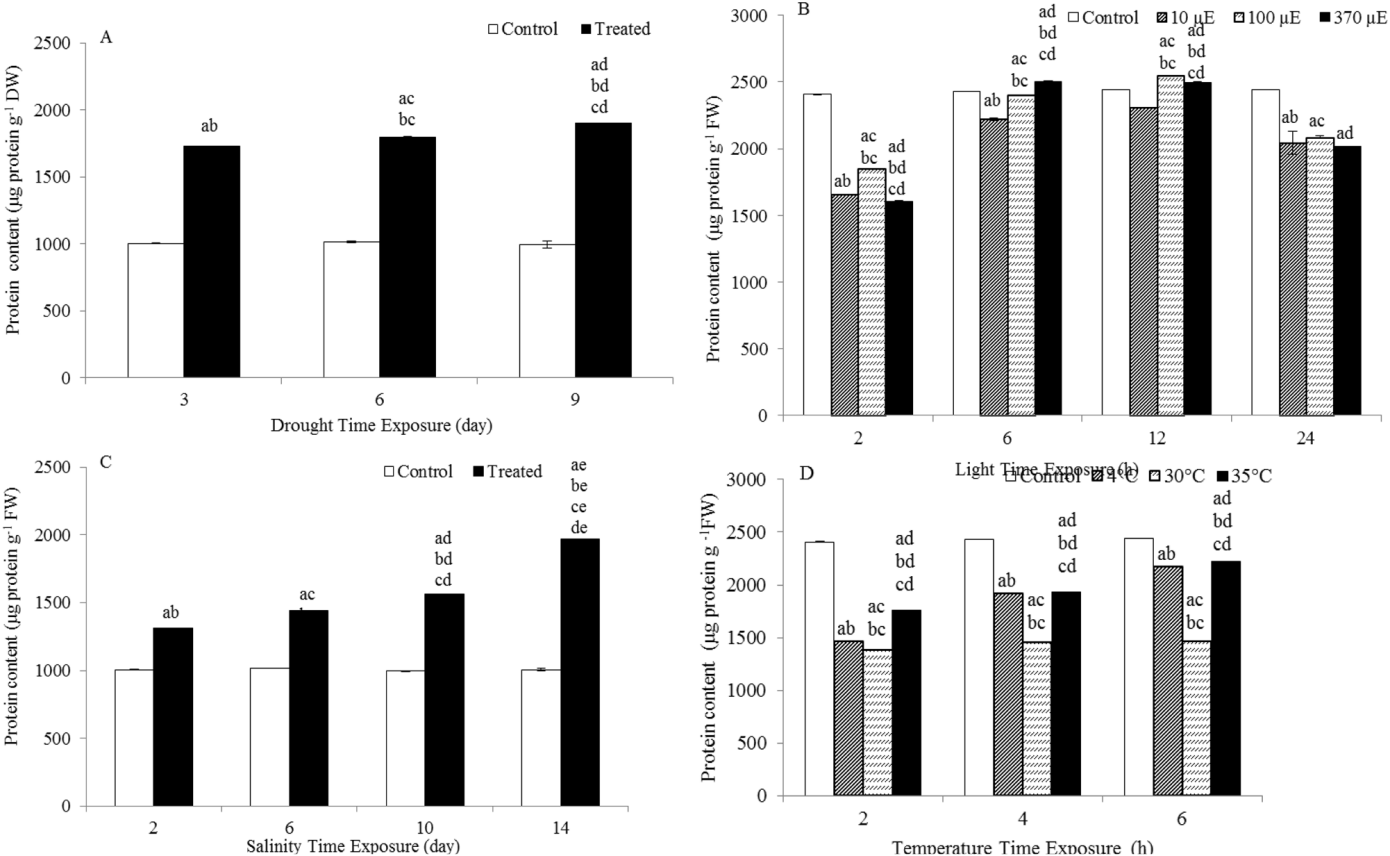
Protein content in the leaves of *L. sativum* subjected to various abiotic stresses. (**A**) Drought. (**B**) Light intensity. (**C**) Salinity. (**D**) Heat. The effect of different abiotic stresses on protein content at different time points compared with the protein content in the controls. Data represent mean values ± SD; n = 3. The significance of differences was calculated using Tukey’s test, with *P* < 0.05 indicating a significant difference.

The protein content was significantly increased by 1.7-, 1.8- and 1.9-fold after 3, 6 and 9 days of drought treatment, respectively, compared to the protein content in the controls. The protein content in salinity-treated plants was significantly increased by 1.3-, 1.4-, 1.6- and twofold after 2, 6, 10 and 14 days of treatment, respectively, compared to the protein content in the controls, as shown in Fig. 5.

### Effects on total APX specific activity

**Figure 6:**
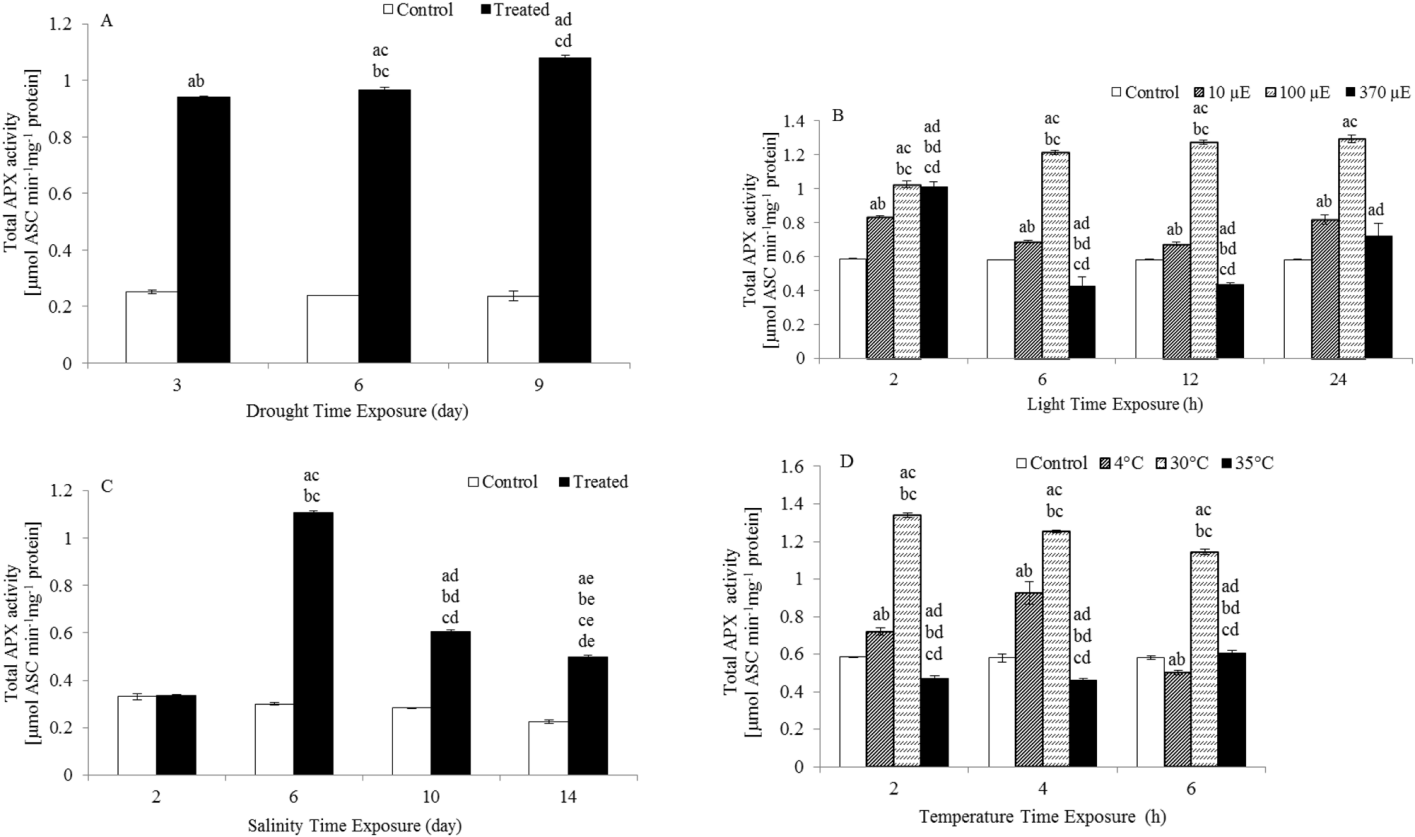
Total APX activity in the leaves of *L. sativum* subjected to various abiotic stresses. (**A**) Drought. (**B**) Light intensity. (**C**) Salinity. (**D**) Heat. The effect of different abiotic stresses on APX activity at different time points compared with the APX activity in controls. Data represent mean values ± SD; n = 3. The significance of differences was calculated using Tukey’s test, with *P* < 0.05 indicating a significant difference.

A significant increase in APX specific activity was observed in drought-, light-, salinity- and temperature-stressed plants compared to the APX specific activity in control plants (Fig. 6). Drought treatment resulted in a 3.7-, 4- and 4.6-fold increase in total APX specific activity after 3, 6 and 9 days of treatment, respectively. Exposure to 10 µE light increased the total APX specific activity by 1.4-, 1.2-, 1.2- and 1.4-fold after 2, 6, 12 and 24 h of treatment, respectively, compared to the APX specific activity in control plants. Exposure to 100 µE light increased the APX specific activity by 1.8-, 2.1-, 2.2- and 2.2-fold after 2, 6, 12 and 24 h of treatment, respectively. Furthermore, exposure to 370 µE light increased the APX specific activity by 1.4- and 1.2-fold after 2 and 24 h of treatment, respectively. Salinity treatment resulted in a 3.7-, 2.1- and 2.2-fold increase in APX specific activity after 6, 10 and 14 days of treatment, respectively. In addition, exposure to 4 °C caused an increase in APX specific activity of 1.2- and 1.6-fold after 2 and 4 h of treatment, respectively. Finally, exposure to 30 °C for 2, 4 and 6 h increased APX specific activity by 2.3-, 2.2- and twofold, respectively, compared to the APX specific activity in the controls.

## Discussion

The currently reported results of *Lepidium sativum* (cress) plant in response to most of the applied stressors (drought, salinity, low light quantity and temperature) indicated common biochemical reactions observed as increment in all the studied stress indicators. However, the exposure of the plant to low light quantity and high temperature exhibited some uncommon reactions. Under low light, anthocyanin, carotenoids and H_2_O_2_ contents increased. On the contrary, MDA, protein and APX decreased. The most divergent responses were observed in the case of low and high-temperature. Under the latter stressor, anthocyanin and carotenoids contents, MDA and protein content decreased, while under low temperature anthocyanin and carotenoids contents increased and the other indices were decreased.

Similar to most of our results, it was reported that the levels of endogenous anthocyanins in foliage are stimulated by different abiotic factors^10,19^. However, contrast results of elevated anthocyanin content in response to long-term exposure (6 weeks) to temperature^20^ or to the synergetic effect of high temperature and duration of cultivation^21^ was reported. Chalker–Scott^19^ also reported different finding from the present study where low temperatures in the absence of either visible or ultraviolet B (UVB) light prevent anthocyanin biosynthesis. Anthocyanin accumulation has been proposed to have a role in protection against photoinhibition, in which anthocyanins serve as osmotically active solutes and antioxidants for ROS in addition to their function as a UV screen^22–25^. In addition, accumulated anthocyanins might act as signaling markers of plant stress induced by salt^26^ enhancing peroxidase activity and anthocyanin content^26,27^. Similarly, elevated carotenoids levels allow plants to cope with oxidative stress^28,29^ by scavenge ROS to prevent the oxidation of membrane lipids, and ultimately mitigate oxidative stress. Thus, plants can tolerate stressful conditions by maintaining a higher or invariable level of total carotenoids. The results of the present study regarding carotenoids accumulation are in agreement with those of previous studies^30–32^ on plant acclimations to stress.

Increased production of ROS is one of the earliest responses of plant cells to abiotic stress^33^. Among ROS, H_2_O_2_ has been found to trigger a variety of plant responses^33,34^. Drought and light stress found to be linearly induces H_2_O_2_ accumulation^35–37^. H_2_O_2_ is versatile and plays a series of roles that range from orchestrating physiological processes to the stress response^37,38^. In the present study, our results on the effects of salinity stress on the H_2_O_2_ content in leaves are in agreement with those from previous reports^39^. H_2_O_2_ can induce antioxidant enzymatic defenses to reduce the deleterious effects of salinity since H_2_O_2_ is a signaling molecule that mediates crosstalk between signaling pathways and contributes to protection against other sources of stresses^40^.

Plants tolerance to temperature stress depending upon the plant type, duration and intensity of the stress have been reviewed by Awasthi et al.^41^. Furthermore, the variations in the levels of MDA and H_2_O_2_ levels might be temperature-specific ^42^, which might explain the reduction of the levels of these molecules during low and high temperature stress. Lipid peroxidation induced by a variety of stresses is involved in diverse signaling processes to protect plants from oxidative stress^43–46^. The overproduction of ROS increases the content of MDA, which is an indicator of oxidative damage^47^. Our results show a correlation between H_2_O_2_ accumulation and the increase in MDA level in response to drought, salinity and light stress. Salt stress results in extensive lipid peroxidation, which is often used as a marker of stress-induced cellular damage. Therefore, plants that tolerate salt stress are better protected from leaf oxidative damage^48,49^. The significant change in protein content in response to the severity of abiotic stress might be a mechanism to enhance plant stress tolerance through the increased abundance of proteins involved in energy production, amino acid synthesis, protein synthesis and the antioxidant defense system^50–53^. Our results showing changes in protein content in response to salt stress are consistent with those previously reported findings, in which an increased leaf protein concentration was directly associated with stress tolerance^54,55^. Gülen and Eris^56^ reported similar results showing that the total protein content is decreased by heat stress. This reduction in total protein content might be due to the occasional production of specific proteins to reduce stress severity, such as peroxidases and ROS scavenger enzymes, as an acclimation response^57^.

APX is ROS-scavenging enzyme^58^, that minimize the effects of oxidative stress by cooperating with other proteins to maintain the integrity of photosynthetic membranes under oxidative stress through direct scavenges ROS or producing a nonenzymatic antioxidant^58^. Because of this, APX plays a key role in the acclimation of plants to stress^59^ via the metabolism of H_2_O_2_ which leads to increasing plant cellular tolerance to oxidative stress^38,60^. Regulation of the activities and levels of APX isoenzymes and other antioxidant enzymes offers additional stress defense capabilities^11,61^. In agreement with our results, previous studies on the influence of salt stress, high and low temperatures reported increment in total specific APX activity, which might help in the reduction of stress severity as an acclimation response^56,57,62^. In particular, at 30 °C our findings are in agreement with the observations of Kumar et al.^42^ for the changes in total specific APX activity to different temperatures. According to Pandey et al.^63^, H_2_O_2_ at low levels acts as a secondary messenger in initiating scavenging system including increasing APX activity. The considerable increase in APX activity could not stop the deleterious effects of heat stress but reduced stress severity as an acclimation response.

The current finding suggests that *L*. *sativum* launch common protective pathways under drought, salinity, and high light stresses, while another protective mechanism could be responsible for increasing the plants tolerance towards high temperature and low light. Further investigation is required to clarify the cascade of biochemical changes starting from the molecular level.

## Abbreviations

*APX*: Ascorbate peroxidase
*MDA*: Malondialdehyde
*ROS*: Reactive oxygen species
